# Transporter *TAP1*-637G and Immunoproteasome *PSMB9*-60H Variants Influence the Risk of Developing Vitiligo in the Saudi Population

**DOI:** 10.1155/2014/260732

**Published:** 2014-12-07

**Authors:** Nasser Attia Elhawary, Neda Bogari, Essam Hussien Jiffri, Mona Rashad, Abdulhamid Fatani, Mohammed Tayeb

**Affiliations:** ^1^Department of Medical Genetics, Faculty of Medicine, Umm Al-Qura University, P.O. Box 57543, Mecca 21955, Saudi Arabia; ^2^Department of Molecular Genetics, Medical Genetics Center, Faculty of Medicine, Ain Shams University, Cairo 11566, Egypt; ^3^Department of Medical Laboratory Technology, Faculty of Applied Medical Sciences, King Abdulaziz University, Jeddah 21589, Saudi Arabia; ^4^Department of Pediatrics, Al-Qatif Central Hospital, Dammam 31911, Saudi Arabia; ^5^Department of Pediatrics, Faculty of Medicine, Ain Shams University, Cairo 11566, Egypt; ^6^National Guard Hospitals, Faculty of Medicine, King Saud bin Abdulaziz University for Health Sciences, Riyadh 11564, Saudi Arabia

## Abstract

We evaluated whether *TAP1*-rs1135216 (p.637D>G) and *PSMB9*-rs17587 (p.60R>H) were significantly associated with the risk and severity of vitiligo among Saudi patients. One hundred seventy-two subjects were genotyped for the *TAP1*-rs1135216 and *PSMB9*-rs17587 variants using endonuclease digestions of amplified genomic DNA. The *TAP1*-rs1135216 and *PSMB9*-rs17587 mutant alleles were strongly associated with vitiligo, with odds ratios showing five fold and two fold risks (*P* < 0.0001 and *P* = 0.007, resp.). In *TAP1*-rs1135216, the 637G mutant allele was more frequent in cases (74%) than in healthy controls. In cases, the 60H mutant allele *PSMB9*-rs17587 was less frequent (42%) than the wild-type 60R allele (58%). Vitiligo vulgaris was the most common type of disease, associated with the DG (55%) and GG (46%) genotypes for rs1135216 and with the RH genotype (59%) for rs17587. The heterozygous 637DG and 60RH genotypes were each linked with active phenotypes in 64% of cases. In conclusion, the *TAP1*-rs1135216 and *PSMB9*-rs17587 variants are significantly associated with vitiligo, and even one copy of these mutant alleles can influence the risk among Saudis. Vitiligo vulgaris is associated with genotypes containing the mutant G and H alleles.

## 1. Background

Vitiligo is characterized by skin depigmentation due to a lack of melanocytes in the dermis or the inability to produce melanin. The depigmentation takes the form of circumscribed, white macules in the skin that form when the melanocytes in the epidermis destruct [1]. Worldwide prevalence of vitiligo ranges from 0.09% to 8% [2]: 0.14% in Russia, 0.38% in Caucasians [2], 0.22–1.22% in Egypt [3, 4], 1–2.5% in the United States and Japan [5], 4% in Mexico [6], and 8% in India [7]. The prevalence among randomly selected female students in an eastern Saudi province has been recorded as 0.4% [8]. Despite the low prevalence of vitiligo in Saudi Arabia, the disease represents a great burden on the social and psychological well-being of the Saudi community [9].

Studies have shown that autoimmune processes participate in the pathogenesis of vitiligo [10]. Besides, previous studies suggest that nitric oxide, as a free radical initiator, has been involved in the inhibition of cell proliferation, differentiation, and apoptosis and, thus, may also contribute to the pathogenesis of various autoimmune diseases [11]. Other theories that have been proposed to explain the pathomechanisms of vitiligo include neural, radical, self-destruction, and inherent-defect theories [12]. Although some of them may be relevant to vitiligo, none of them explain the pathological mechanism of the disease perfectly.

Several candidate genes have been linked to vitiligo [13], such as genes involved in the leukocyte antigen system (HLA, MIM 615161), cytotoxic T lymphocyte-associated 4 (CTLA4, MIM 123890), tumor necrosis factors (TNF*α*, MIM 191160), and autoimmune regulators (AIRE gene, MIM 607358) [14]. Transporter associated with antigen processing (*TAP*) genes are encoded in the MHC-II region of the human HLA locus (MIM 615161). TAP is composed of two integral membrane proteins, TAP1 and TAP2, which assemble into a heterodimer that results in a four-domain transporter. TAP1 functions by providing candidate peptides to the MHC-I molecules within the peptide-loading complex and by transporting antigen peptides from the cytoplasm into the endoplasmic reticulum [15]. Proteasomes are responsible for degrading short-lived cytoplasmic proteins into peptides [16]. Among its 28 subunits, the 20*S* proteasome includes two subunits known as* PSMB8* (*LMP7*) and* PSMB9* (*LMP2*).

*TAP* and the immunoproteasome* PSMB* have been reported to be associated with several autoimmune diseases [16], such as celiac disease [17], Sjögren's syndrome [18], type 1 diabetes [19, 20], juvenile rheumatoid arthritis [18], and multiple sclerosis [21]. A few case-control studies have also shown genetic variants of* TAP* and* PSMB* to be associated with vitiligo in ethnic Caucasians, suggesting a possible role in the antimelanocyte autoimmune response involved in the disease [22]. The genetic variants* TAP1*-rs5735883 and* PSMB8*-rs37360 have been studied in Saudi vitiligo cases [23], but no significant associations have been found.

Although a large amount of genetic information is available on vitiligo, most of the reports are from Western populations. Only 14 articles have focused on vitiligo in Middle East populations; two of them are from Saudi Arabia [24, 25]. Here, we report the findings of a study to further investigate the associations between* TAP1*/*PSMB* genetic variants and vitiligo in Saudi patients and to evaluate the influence of genotypes on risk and severity of disease. We specifically focused on the* TAP1*-rs1135216 (p.637D>G) and* PSMB9*-rs17587 (p.60R>H) mutant alleles.

## 2. Subjects and Methods

### 2.1. Study Population

A group of 86 Saudi patients with vitiligo were selected from dermatologic outpatient clinics of different provinces in Saudi Arabia for molecular study at the Medical Genetics Laboratories, Medical College, Umm Al-Qura University. After giving their informed consents, patients were interviewed and evaluated to confirm the diagnosis of vitiligo. The information gathered from the patients included age at disease onset, gender, clinical history of the patients and their relatives, consanguineous status (if present), history of other autoimmune disorders, previous treatment (if any), and types and distribution of vitiligo lesions in the patient and pedigree. Patients who had been exposed to any therapy in the past six months were excluded from the study.

Classic subtypes of vitiligo were classified as* focal* (if there was one or more maculae in a nonsegmented pattern),* vulgaris* (if there was symmetric or asymmetric distribution of maculae in one or more areas),* segmental* (if there were unilateral depigmented macules that did not cross the midlines),* acral*/*acrofacial* (if there was loss of skin color on tips of fingers and toes, the anogenital area, and the lips and “around the eyes” area of the face), and* universalis* (complete or >80% skin depigmentation). The phenotype of the disease was classified as active (if it was progressive, or if new maculae had appeared in the past six months) or stable. None of the healthy subjects (*n* = 86) showed clinical evidence or a family history of vitiligo or of any other autoimmune disorder. The protocols used in the study were approved by the Biomedical Ethics Committee, Faculty of Medicine, Umm Al-Qura University.

### 2.2. DNA Isolation

Genomic DNA samples were extracted from peripheral blood (200 *μ*L) using the QIAamp DNA blood kit (Qiagen, Hilden, GmbH, Germany). In some cases, DNA was prepared* in situ* by gentle scraping of the buccal mucosa for 30 sec using a cytobrush [26]. The cells obtained were treated directly with diluted NaOH solution, heated, and neutralized with Tris-Cl, pH 8.0. A 2.5 mL volume of buccal cells typically sufficed for amplification by polymerase chain reaction (PCR).

### 2.3. Genotyping of the rs1135216 and rs17587 Loci

Genomic DNA was added to a 25 *μ*L reaction volume containing 50 mM KCl, 10 mM Tris-Cl, pH 8.3, 1.5 mM MgCl_2_, 60 mM of each dNTP, and 0.25 units of* Taq* DNA polymerase (BIORON, GmbH, Germany). Previously reported primers for the rs1135216 single nucleotide polymorphism (SNP)—the forward primer 5′-CTC ATC TTG GCC CTT TGC TC-3′ and the reverse 5′-CAC CTG TAA CTG GCT GTT TG-3′—and for the rs17587 SNP—the forward primer 5′-GTG AAC CGA GTG TTT GAC AAG C-3′ and the reverse 5′-GCC AGC AAG AGC CGA AAC AAG-3′ [22]—were synthesized (Metabion Co., GmbH, Germany). PCR samples were subjected to 35 cycles on PCR Engine Dyad (Bio-Rad Laboratories Inc., Hercules, CA) with annealing at 58°C for 30 sec. To genotype these SNPs, the rs1135216 and rs17587 PCR amplicons were incubated with the* Acc*I and* Hha*I enzymes (New England Biolabs, Beverly, MA), respectively, at 37°C for 2 h. The fragments were separated on a 3% MetaPhor agarose gel (BMA, Rockland, ME) using ethidium bromide staining and viewed under a UV transilluminator (G-Box, SynGene, Frederick, MD).

The 637D allele of* TAP1* remained uncut (165 bp), but the 637G allele was cleaved into two fragments (136 and 29 bp). Similarly, the 60H allele of* PSMB9* remained uncut (252 bp), but the 60R allele was cleaved into two fragments (212 bp and 40 bp). A positive control was used for each polymorphism. Each sample was run in duplicate. The genotypes of all samples were reassessed twice to confirm the results and ensure reproducibility. Some suspected genotypes were validated by purifying the PCR products using automated Agencourt AMPure XP kit (Beckman Coulter, Canada) and genotyping using Genetic Analyzer 3500 (ABI, Life Technologies, USA).

### 2.4. Data Analysis

Statistical analysis was performed using SPSS 20.0 (SPSS Inc., Chicago, IL). The data were presented as means ± standard deviations. Student's* t*-tests and *χ* ^2^-tests were used to compare continuous and categorical variables. Multivariate logistic regression analysis was performed to assess the contributions of* TAP1*-rs1135216 and* PSMB9*-rs17587 alleles and other independent risk factors to study the severity of vitiligo disease. A *P* value of <0.05 was considered statistically significant. Odds ratios (ORs) with 95% confidence intervals (CIs) were also calculated using the Mantel-Haenszel method. The distribution of the control genotypes was checked for Hardy-Weinberg equilibrium using the *χ* ^2^-test (http://www.oege.org/software/hwe-mr-calc.shtml).

We used G^*^Power software (Germany, version 3.1.5, http://www.psycho.uni-duesseldorf.de/abteilungen/aap/gpower3/download-and-register/) to perform* a priori* power analysis to estimate sufficient sample sizes to achieve adequate power for *z*-testing of two independent proportions.* A priori* sample-size estimations were performed using known information on the common allele frequencies in vitiligo patients, a criterion probability of *α* = 0.05, and a power sensitivity of 80%. The prevalence of vitiligo in the studied population was assumed to be 50%, with a case-control ratio of 1.

## 3. Results

### 3.1. Characteristics of the Study Population

Table 1 shows selected demographic and clinical characteristics of the 86 vitiligo patients. The mean age at onset was 11.5 years and at examination was 22.0 years. The gender distribution of the patients was 1 : 1. No significant differences (*P* < 0.05) were found between cases and controls with regard to age and sex.

About 40% of the patients had a positive family history and 16% had consanguinity. Most had an active phenotype of depigmented patches (67%), but 33% had a stable phenotype. All patients had the generalized type of vitiligo, and 50% suffered from sensitivity to the sun. Vitiligo cases with hypothyroidism differed significantly (16.3%; *P* < 0.0001) from cases without the thyroid pathology, but cases with diabetes mellitus type 1 did not differ significantly (5.8%; *P* = 0.73) from cases without diabetes. Relatively few cases showed early graying of hair (29%).

### 3.2. Allele Frequencies and Genotype Distribution

Tables 2(a) and 2(b) illustrates the allele frequencies and genotype distribution of the* TAP1*-rs1135216 and* PSMB9*-rs17587 SNPs. The genotype frequencies deviated from the Hardy-Weinberg equilibrium for the rs1135216 SNP (*P* < 0.05) but satisfied this equilibrium for rs17587 (*P* > 0.05) in the healthy controls; the difference between the expected and observed values for the control genotypes was not significant (*P* < 0.05). The allele frequencies of the two SNPs were strongly associated with vitiligo, with ORs showing fivefold and twofold risks, respectively (OR = 5.2, *P* < 0.0001 for rs1135216 and OR = 1.9, *P* = 0.007 for rs17587). The 637G mutant allele of* TAP1* was more frequent in cases (74%) than in healthy controls (36%). Among patients, the 60H mutant allele of* PSMB9* was less frequent (42%) than the 60R wild-type allele (58%).

As for genotype distribution, none of the cases had the 637DD genotype and none of the controls had the 637GG genotype. When we focused on the distribution of genotypes containing the mutant alleles (DG+GG and RH+HH), we found highly significant differences between cases and controls (*χ* ^2^ = 16.2, *P* < 0.0001 for* TAP1*-rs1135216 and *χ* ^2^ = 4.5, *P* = 0.03 for* PSMB9*-rs17587).

### 3.3. Stratified Analysis of the Genetic Variants and Clinical Types of Vitiligo

We investigated the effects of the rs1135216 and rs17587 variants on the clinical types of vitiligo (Table 3). Vitiligo vulgaris (VV) was the most common type of disease among the Saudi patients (51%), followed by focal vitiligo (FV, 21%) and acral/acrofacial vitiligo (AV, 19%). Segmented vitiligo (SV) and universalis vitiligo (UV) were the least common (2% and 7%, resp.). Among the VV cases, 55% had the 637DG genotype and 46% had the 637GG genotype (both containing the mutant G allele); 59% of the VV cases had the 60RH genotype. The mutant 637GG genotype was most frequent in FV (78%) and VV (67%) cases. The heterozygous 60RH genotype was most frequent in AV (50%), FV (56%), and VV (59%) cases. Despite the lower frequency of SV cases (2%) in our population, all these cases had the heterozygous 637DG and 60RH genotypes.

As for genotype-phenotype correlation, 64% of cases with the heterozygous 637DG genotype and 64% of cases with the heterozygous 60RH genotype were linked with active phenotypes (Table 4). There were no associations between the D637G and R60H genotypes and active/stable phenotypes (OR = 0.66, *P* = 0.83 for D637G and OR = 1.2, *P* = 0.75 for R60H). Moreover, there was a significant association between the active and stable phenotypes and the* TAP1* polymorphism (OR = 2.70, *P* = 0.01) but not between the active and stable phenotypes and the* PSMB9*-R60H polymorphism (OR = 1.0, *P* = 0.9).

## 4. Discussion

This study investigated the distribution of two biallelic variants of the* TAP1*-rs1135216 and* PSMB9*-rs17587 loci, corresponding to amino acid positions 637 and 60, in a group of 86 Saudi patients and 86 healthy controls. The* TAP1* 637G and* PSMB9* 60H mutant alleles were found to significantly increase the risk of vitiligo (fivefold and twofold, resp.) when compared with wild-type alleles (*P* < 0.0001 and *P* < 0.007, resp.).

The* TAP1* 637G and* PSMB9* 60H mutant alleles were found to significantly increase the risk of vitiligo (fivefold and twofold, resp.) when compared with wild-type alleles (*P* < 0.0001 and *P* < 0.007, resp.). An earlier study in Caucasians revealed a significant association between vitiligo and* TAP1*-rs1135216 (*P* = 0.0034) but a nonsignificant result between vitiligo and* PSMB9*-rs17587 (*P* = 0.11) [22].

Our study showed that VV (the major subtype of vitiligo in our sample) was remarkably associated with the mutant 637G and mutant 60H alleles of the polymorphisms we studied. There were no significant associations between the phenotypes of vitiligo (i.e., active, stable) and either the D637G or the R60H polymorphic marker (*P* = 0.83 and *P* = 0.75, resp.). It was clear that only the mutant 637G allele significantly affected the progression or the stability of vitiligo among our cases (*P* = 0.01).

The present study also identified some interesting clinical findings. Type 1 diabetes mellitus was seen in 5.8% of our vitiligo cases, although no significant association was found between the two (*P* = 0.73). However, the frequency of type 1 diabetes in our vitiligo cases was twofold higher than that reported in Caucasians [27], Turks [28], and Jordanians [29]. The higher frequency of diabetes might be due to either the higher incidence of diabetes among the Saudi community or the interaction of multiple genes affecting both vitiligo and diabetes. Moreover, Somorin and Krahn [30] found vitiligo to be associated with diabetes mellitus in 5% of vitiligo cases but mostly in the form of type 2 diabetes.

Patients with vitiligo have a strong predisposition to develop other autoimmune conditions, particularly those affecting the thyroid [31]. Indeed, the prevalence of autoimmune thyroid disease in vitiligo cases has been estimated at 14%, and the risk of developing this type of disease has been reported to be 2.5-fold higher for patients with vitiligo than for individuals without vitiligo [31]. The prevalence of thyroid pathology in Saudis with vitiligo (16.3%, *P* < 0.0001) was higher in our study than that reported in Turkish (4.4%) [28] or Chinese (6.8%) [32] individuals with vitiligo. In a cohort study of Caucasians, 5.7% of first-degree relatives (parents and siblings only) of vitiligo patients were reported as having clinical autoimmune thyroid disease, which was more than twice the population frequency (*P* < 0.001) [27].

As generally reported, vitiligo affects both sexes equally. Although the age of onset is variable, most patients develop symptoms between 10 and 30 years of age [33]. The mean age of onset in our Saudi cases—11.5 years (range, 2–47 years)—was consistent with a previous Saudi study [34]. Most populations, with the exception of those in Egypt [35], have reported a later mean age of onset, of at least 21 years [22, 27–29]. In line with a low age of onset in Saudi Arabia, more than 55% of the cases in our study had an age of onset under 10 years, and about 89% had one under 20 years.

The proportion of Caucasian families with aggregated cases of vitiligo has been estimated at 20% [36]. In the Saudi community, about 54% of marriages are consanguineous [37], and the incidence of vitiligo is proportionally higher, 16% among our Saudi sample, with positive family history reaching 40%. An epidemiological Saudi study from the Northern province of Arar has recently reported that consanguinity highly increases the incidence of the disease, with a frequency of 65% (45 out of 69 patients) [38]. Consanguinity, which is relatively frequent in the Middle East and some other parts of the world, is usually socioeconomically and culturally motivated and can be genetically harmful.

The present study had some limitations. First, our* post hoc* statistical analysis for the rs17587 SNP revealed a power of 49% among our 172 participants. Recruiting more participants (i.e., 368 participants, aiming for a power of 80%) in both case and control groups within a reasonable time frame from a few outpatient clinics would have been difficult. Hence, replication of our results through larger, multicenter genetic association studies will be important. Second, we restricted this study to two polymorphic markers that we hypothesized to be significantly associated with the disease. Spritz and colleagues [39] have previously described genome-wide linkage analyses among multiplex vitiligo families, aimed at detecting the locations of genes that contribute to the risk of vitiligo [29, 39, 40]. These studies identified several linkage signals that met formal genome-wide criteria for “significant” linkage [41]. More linkage studies based on a whole-genome approach instead of a single or a few candidate genes may be useful for discovering new genes associated with susceptibility to vitiligo.

## 5. Conclusions

This is the first study, to our knowledge, to report associations between the allelic variants of rs1135216 and rs17587 loci and vitiligo in the Saudi population. We found that one copy of the mutant alleles* TAP1* 637G and* PSMB9* 60H can influence the development of, or induce the progression or appearance of, new depigmented lesions. The identification of new susceptibility genes has opened new avenues for exploring the underlying disease mechanisms for vitiligo.

## Figures and Tables

**Table 1:** Demographic and clinical features of vitiligo patients (*n* = 86).

Characteristic	Number (%)^a^	*z* (*P* value)^b^
Average age (mean ± SD)		
At onset (range)	11.5 ± 10.1 y (2–47 y)	6.0 (*P* < 0.0001)^c^
		(9.3–13.7)
At examination (range)	22.0 ± 12.6 y (2–50 y)	12.5 (*P* < 0.0001)^c^
		(19.3–24.7)
Ratio of women : men	1 : 1	
Family history	34/86 (39.5)	12.3 (<0.0001)
Consanguinity	14/86 (16.3)	4.8 (<0.0001)
Progressive/stable^d^	48 (66.7)/24 (33.3)	24.0 (<0.0001)
Sensitivity to sun	43/86 (50)	19.1 (<0.0001)
Thyroid pathology (hypo)	14/86 (16.3)	4.8 (<0.0001)
Diabetes mellitus type 1	5/86 (5.8)	0.34 (0.73)
Early graying of hair	24/86 (27.9)	10.3 (<0.0001)
Halo nevi^e^	5/18 (27.8)	10.0 (<0.0001)

^a^Numbers in parentheses are expressed in percentages unless otherwise stated.

^b^All values are expressed in terms of *z*-test unless otherwise stated.

^c^Values are expressed in terms of *t*-test.

^d^14 cases could not be clinically followed up.

^e^Only 18 of 86 cases were investigated for halo nevi.

**(a):** 

Allele	Vitiligo patients	Controls	OR	*z* (*P* value)	95% CI
	*n* (frequency)	*n* (frequency)			
D	44 (0.26)	110 (0.64)	5.2	7.0 (<0.0001)	3.2–8.2
G	128 (0.74)	62 (0.36)			
R	100 (0.58)	124 (0.72)	1.86	2.7 (0.007)	1.2–2.9
H	72 (0.42)	48 (0.28)			

**(b):** 

Genotype	*n* (%)	*n* (%)	*χ* ^2^ (*P* value)
*TAP1*-D637G:			
DD	0 (00.0)	24 (27.9)	16.2 (<0.0001)
DG	44 (51.2)	62 (72.1)	
GG	42 (48.8)	0 (00.0)	
(DG + GG)	86 (100)	62 (72.1)	
*PSMB9*-R60H:			
RR	26 (30.2)	44 (51.1)	4.5 (0.03)
RH	48 (55.8)	36 (41.9)	
HH	12 (14.0)	6 (7.0)	
(RH + HH)	60 (69.8)	42 (48.9)	

**Table 3:** Stratification of *TAP1*-D637G and *PSMB9*-R60H genotypes associated with different clinical types of vitiligo.

Vitiligo type	Number of cases (%)	*TAP1*-D637G			*PSMB9*-R60H		
		*n* (%)			*n* (%)		
		DD	DG	GG	RR	RH	HH
VV	44 (51.2)	0 (0.0)	24 (54.5)	20 (45.5)	10 (22.7)	26 (59.1)	8 (18.2)
UV	6 (7.0)	0 (0.0)	4 (66.7)	2 (33.3)	2 (33.3)	2 (33.3)	2 (33.3)
FV	18 (20.9)	0 (0.0)	4 (22.2)	14 (77.8)	6 (33.3)	10 (55.6)	2 (11.1)
AV	16 (18.6)	0 (0.0)	10 (62.5)	6 (37.5)	6 (37.5)	8 (50.0)	2 (12.5)
SV	2 (2.3)	0 (0.0)	2 (100)	0 (0.0)	0 (0.0)	2 (100)	0 (0.0)

AV: acral/acrofacial vitiligo; FV: focal vitiligo; SV: segmental vitiligo; UV: universalis vitiligo; VV: vulgaris vitiligo.

^*^“*n*” is the number of genotypes in *TAP1* or *PSMB9* variants.

**Table 4:** *TAP1*-D637G and *PSMB9*-R60H genotype distribution and allelic frequencies for active versus stable vitiligo phenotypes.

Phenotype	Number of cases (%)	*TAP1*-D637G genotypes^a^			Alleles		*PSMB9*-R60H genotypes			Allele	
		*n* (%)			*n* (frequency)		*n* (%)			*n* (frequency)	
		DD	DG	GG	D	G	RR	RH	HH	R	H
Active	52 (60.5)	0 (0.0)	33 (63.5)	19 (36.5)	33 (0.32)	71 (0.68)	9 (17.3)	33 (63.5)	10 (19.2)	51 (0.49)	53 (0.51)
Stable	34 (39.5)	0 (0.0)	10 (29.4)	24 (70.6)	10 (0.15)	58 (0.85)	5 (14.7)	24 (70.6)	5 (14.7)	34 (0.50)	34 (0.50)

		OR = 0.66, 95% (CI, 0.01–33.9),			OR = 2.70, 95% (CI, 1.2–5.9),		OR = 1.2, 95% (CI, 0.4–4.0),			OR = 1.0, 95% (CI, 0.5–1.8),	
		*z* = 0.21, *P* = 0.83^a^			*z* = 2.5, *P* = 0.01^b^		*z* = 0.3, *P* = 0.75^c^			*z* = 0.1, *P* = 0.90^b^	

CI: confidence interval; OR: odds ratio.

^a^ *TAP1*-D637G genotype differences between progressive and stable vitiligo cases.

^b^ *P* > 0.05 = no significant difference.

^c^ *P* < 0.05 = a significant difference.
